# Low-dose intravenous plus inhaled versus intravenous polymyxin B for the treatment of extensive drug-resistant Gram-negative ventilator-associated pneumonia in the critical illnesses: a multi-center matched case–control study

**DOI:** 10.1186/s13613-022-01033-5

**Published:** 2022-08-08

**Authors:** Jiao Liu, Min Shao, Qianghong Xu, Fen Liu, Xiaojun Pan, Jianfeng Wu, Lihong Xiong, Yueming Wu, Mi Tian, Jianying Yao, Sisi Huang, Lidi Zhang, Yizhu Chen, Sheng Zhang, Zhenliang Wen, Hangxiang Du, TaoWang, Yongan Liu, Wenzhe Li, Yan Xu, Jean-louis Teboul, Dechang Chen

**Affiliations:** 1 Department of Critical Care Medicine, Ruijin Hospital, Shanghai Jiao Tong University School of Medicine, No.197 Ruijin 2nd Road, Shanghai, 201801 China; 2 Department of Critical Care Medicine, The First Affiliated Hospital of Anhui Medical University, Hefei, 230022 China; 3 Department of Critical Care Medicine, Zhejiang Hospital, No.12 Lingyin Road, HangZhou, 310015 China; 4 Department of Critical Care Medicine, the First Affiliated Hospital of Nanchang University, No.17, YongwaiZheng Street, Nanchang, 330006 Jiangxi China; 5 Department of Critical Care Medicine, The First Affiliated Hospital, Sun Yat-Sen University, No. 58 Zhongshan Er Road, Guangzhou, 510010 China; 6 Department of Intensive Care Unit, The Second People’s Hospital of Shenzhen, Futian District, Sungang West Road, Shenzhen, 3002518035 China; 7 Emergency and Critical Care Center, Lishui People’s Hospital, No. 15 Dazhong Road, Lishui, 323000 China; 8 Department of Intensive Care Unit, Huashan Hospital, Fudan University, No. 12 Middle Urumqi Road, Shanghai, 200040 China; 9 Department of Intensive Care Unit, The First People’s Hospital of KunShan, No 91, Qianjin Road, KunShan, 215300 China; 10 Service de Médecine-Intensive Réanimation, Hôpital Bicêtre, AP-HP. Université Paris-Saclay, Inserm UMR 999, Université Paris-Saclay, 94270 Le Kremlin-Bicêtre, France

**Keywords:** Polymyxin B, Inhalation, Extensively drug-resistant Gram-negative bacilli, Ventilator-associated pneumonia, Critical illnesses

## Abstract

**Background:**

The mortality of extensively drug-resistant Gram-negative (XDR GN) bacilli-induced ventilator-associated pneumonia (VAP) is extremely high. The purpose of this study was to compare the efficacy and safety of inhaled (IH) plus intravenous (IV) polymyxin B versus IV polymyxin B in XDR GN bacilli VAP patients.

**Methods:**

A retrospective multi-center observational cohort study was performed at eight ICUs between January 1^st^ 2018, and January 1^st^ 2020 in China. Data from all patients treated with polymyxin B for a microbiologically confirmed VAP were analyzed. The primary endpoint was the clinical cure of VAP. The favorable clinical outcome, microbiological outcome, VAP-related mortality and all-cause mortality during hospitalization, and side effects related with polymyxin B were secondary endpoints. Favorable clinical outcome included clinical cure or clinical improvement.

**Results:**

151 patients and 46 patients were treated with IV polymyxin B and IH plus IV polymyxin B, respectively. XDR *Klebsiella pneumoniae* was the main isolated pathogen (*n *= 83, 42.1%). After matching on age (± 5 years), gender, septic shock, and Apache II score (± 4 points) when polymyxin B was started, 132 patients were included. 44 patients received simultaneous IH plus IV polymyxin B and 88 patients received IV polymyxin B. The rates of clinical cure (43.2% vs 27.3%, *p* = 0.066), bacterial eradication (36.4% vs 23.9%, *p* = 0.132) as well as VAP-related mortality (27.3% vs 34.1%, *p* = 0.428), all-cause mortality (34.1% vs 42.0%, *p* = 0.378) did not show any significant difference between the two groups. However, IH plus IV polymyxin B therapy was associated with improved favorable clinical outcome (77.3% vs 58.0%, *p* = 0.029). Patients in the different subgroups (admitted with medical etiology, infected with XDR *K. pneumoniae*, without bacteremia, with immunosuppressive status) were with odd ratios (ORs) in favor of the combined therapy. No patient required polymyxin B discontinuation due to adverse events. Additional use of IH polymyxin B (aOR 2.63, 95% CI 1.06, 6.66, *p* = 0.037) was an independent factor associated with favorable clinical outcome.

**Conclusions:**

The addition of low-dose IH polymyxin B to low-dose IV polymyxin B did not provide efficient clinical cure and bacterial eradication in VAP caused by XDR GN bacilli.

Keypoints

Additional use of IH polymyxin B was the sole independent risk factor of favorable clinical outcome. Patients in the different subgroups were with HRs substantially favoring additional use of IH polymyxin B. No patients required polymyxin B discontinuation due to adverse events.

**Supplementary Information:**

The online version contains supplementary material available at 10.1186/s13613-022-01033-5.

## Introduction

Ventilator-associated pneumonia (VAP) is a common hospital-acquired infection in intensive care unit (ICU) patients [1]. Potential extensively drug-resistant Gram-negative (XDR-GN) bacilli, such as *Escherichia coli (E. coli)*, *Klebsiella pneumoniae (K. pneumoniae), Pseudomonas aeruginosa (P. aeruginosa)*, and *Acinetobacter baumannii (A. baumannii)* are common pathogens that cause VAP in ICU [2]. The VAP mortality caused by XDR-GN pathogens may be higher than 70% [3]. Besides, the incidence of XDR-GN has increased over recent years [2].

Currently, rare effective antimicrobial strategies are available for XDR GN bacteria. Colistimethate sodium and polymyxin sulfate B have been used as a salvage therapy for XDR GN bacilli causing pneumonia [4]. There are also new emerging cephalosporins/ß-lactamase inhibitors (ceftazidime avibactam, ceftolozane tazobactam, cefiderocol and eravacycline), which are active against XDR GNB causing VAP or VAT in critically ill patients. These new cephalosporins are an alternative option to colistin for treating VAP caused by XDR GNB [5]. However, due to high cost of new cephalosporins/ß-lactamase inhibitors, non-availability in middle-income countries, higher antibiotic pressure in the ICU and higher risk of antibiotic resistance, nebulized colistin is considered to be used for treating XDR GN bacilli causing pneumonia. Some studies have reported poor distribution of colistimethate sodium at the site of infection when administered intravenously, which can negatively affect the treatment of pneumonia and tracheobronchitis caused by multidrug-resistant *P. aeruginosa* [6]. In theory, inhaled route of administration might allow direct access of colistimethate sodium to the site of infection and might limit systemic side effects [7]. Several studies have found that aerosolized colistimethate sodium can improve clinical response and microbiologic eradication rate as adjunctive therapy to intravenous antimicrobials or as monotherapy, but does not affect overall mortality in patients with VAP/ hospital-acquired pneumonia (HAP) [8, 9]. Colistimethate sodium is a prodrug requiring in vivo hydrolysis to release colistin A and B, the active polymyxins E. Polymyxin B is a mixture of polymyxins 1 and 2, two compounds directly active against GNB [10]. Bacterial killing occurs more rapidly with polymyxin B than with colistimethate sodium. In addition, polymyxin B is less nephrotoxic than colistimethate sodium. The purpose of this study was to compare the clinical and microbiological efficacy and safety of inhaled (IH) plus intravenous (IV) polymyxin B versus intravenous IV polymyxin B in VAP patients infected with XDR GN bacilli.

## Methods

### Study design and patient population

This retrospective matching cohort study (1:2) was conducted at eight intensive care units (ICUs) between January 1st 2018, and January 1st 2020. A total of 132 eligible adult patients were included. This study was approved by the Medicine Institutional Review Board of Shanghai Jiaotong University, School of Medicine, Ruijin Hospital. The informed consent was waived.

The inclusion criteria were the following: age 18–80 years old; infected patients were confirmed with monomicrobial VAP caused by XDR *E. coli* or *K. pneumoniae or P. aeruginosa* or *A. baumannii*; at least two consecutive samples on different days (time interval at least 24 h) showed the presence of XDR GN bacilli from bronchial secretions or bronchoalveolar lavage samples; at least 6 doses of inhaled or intravenous polymyxin B usage. Patients who were younger than 18 years or older than 80, had multi-microbial VAP, cystic fibrosis or lung transplantation, were excluded.

### Groups and matching criteria

Patients were divided into two groups: IV polymyxin B group and IH + IV polymyxin B group. All eligible patients were given at least 3 days of intravenous polymyxin B therapy. In addition, the IH + IV polymyxin B group received at least 6 doses of inhaled polymyxin B. To avoid the severe side effects caused by inhaled polymyxin B, inhaled glucocorticoid and bronchodilator were given 30 min before treatment. Furthermore, a vibrating mesh nebulizer was chosen to improve the atomization performance of inhaled polymyxin B. The practice of aerosol delivery of polymyxin B in the department of critical care medicine, Ruijin hospital north was according to *Ehrmann *et al. protocol [11]*.* Standard atomization process was applied in six centers. Moderate-to-deep sedation was administered before starting nebulization. Vibrate mesh nebulizer was placed in the upstream of inspiratory limb, volume-controlled mode was chosen with constant inspiratory flow. End-inspiratory pause was set of 20% of duty cycle. Humidification system was removed during inhalation and resumed at the end of nebulization. Expiratory filter was changed after each nebulization. Patients in the two treatment groups were matched based on the day of data of initiation of polymyxin B therapy.

### Endpoints

The primary endpoint of the present study was clinical cure of VAP. The favorable clinical outcome, microbiological outcome, VAP-related mortality, all-cause in-hospital mortality, VAP-related mortality within 28 days after initiation of polymyxin B, and side effects related to polymyxin B were selected as secondary endpoints. The primary outcome and all-cause in-hospital mortality were also assessed in subgroups (medical *vs.* surgical; low *vs.* high sequential organ failure assessment (SOFA)score; different responsible pathogens; with *vs.* without bacteria, with *vs.* without immunosuppressive status).

### Data collection

The following data were extracted from medical records: age, sex, history of antibiotic and glucocorticoids use within 30 days, complications, etiology, SOFA score, Acute Physiology, and Chronic Health Evaluation (APACHE II) score, microbiological diagnosis of VAP, antimicrobial susceptibility, dose and duration of polymyxin B therapy, duration of ICU stay, duration of mechanical ventilation, clinical outcomes, microbiological outcomes, and drug-related side effects. Data for VAP-related mortality and all-cause mortality during hospitalization, and VAP-related 28-day mortality were also extracted.

#### ***Definition***

VAP was confirmed if pneumonia occurred more than 48 h after endotracheal intubation and mechanical ventilation [12]. Pneumonia was defined based on clinical suspicion (with at least two of the following clinical infectious evidence): new lung infiltration confirmed by chest radiological examination (X-ray or CT scan) the new onset of fever; an oral temperature > 38 °C or < 36 °C; peripheral white blood cell count > 12 × 10^9^/L or < 4 × 10^9^/L, with or without nuclear-left shift; new-onset or aggravation of existing respiratory symptoms, such as purulent sputum, with or without chest pain and confirmed by a positive quantitative results for a respiratory sample (significant threshold ≥ 10^4^ colony-forming units [CFU]/ ml) for bronchoalveolar lavages, ≥ 10^6^ CFU/ml for endotracheal aspirations and ≥ 10^7^ CFU/ml for noninvasive sputum samples). Septic shock was defined based on the Sepsis-3 definition [13].

The microbiological diagnosis was identified using regular biochemical methods. In vitro antimicrobial susceptibility tests were performed based on the criteria adopted at the Clinical and Laboratory Standards Institute. The minimal inhibitory concentration (MIC) was used to evaluate drug resistance. XDR Gram-negative bacilli were defined as bacilli that were only susceptible to polymyxins, including *E.coli*, *K. pneumoniae, P. aeruginosa,* and *A. baumannii* based on laboratory testing [14].

Clinical outcome was classified as clinical cure (*i.e.,* the disappearance of infection-related symptoms and signs by the end of polymyxin B treatment), clinical improvement (*i.e.,* improved infection-related symptoms and signs by the end of polymyxin B treatment compared with before polymyxin B treatment), clinical failure (*i.e.,* persistence or worsening of presenting infection-related symptoms and/or signs during polymyxin B treatment or died). Recurrence of infection was defined as the emergence of infection symptoms (*e.g.,* fever, shortness of breath), laboratory measures indicative of bacterial infection (*e.g.,* C-reactive protein, procalcitonin, white blood cell count) within 72 h after discontinuation of polymyxin B, in the absence of other infection foci. Favorable clinical outcome included clinical cure or clinical improvement, and unfavorable clinical outcome included clinical failure or recurrence [15]. Two physicians, who were blinded to the treatment, independently analyzed clinical outcomes. The reviewers evaluated the data again to achieve a consensus decision if disagreements about clinical outcomes occurred (about 8% of patients).

Bacteriological outcome was defined as elimination of the target XDR GN bacilli (*i.e.,* no growth of the target pathogen in the lower respiratory specimens before discharge), persistence of the target XDR GN bacilli (*i.e.,* persistent existence of the target pathogen with infection-related symptoms and signs), colonization (*i.e.,* persistent existence of the target pathogen without infection-related symptoms and signs), and recurrence of the target XDR GN bacilli (*i.e.,* regrowth of the same pathogen with infection-related symptoms and signs after discontinuation of polymyxin B during hospitalization) [15].

VAP-related mortality was defined as death due to the deterioration of signs of pneumonia and as death due to respiratory failure or septic shock.

Nephrotoxicity is the main side effect caused by the systematic administration of polymyxin B. Diagnosis of nephrotoxicity was based on risk, injury, failure, loss, end-stage kidney disease (RIFLE) criteria [16]. At least over 1.5-fold increase in serum creatinine levels or a 25% decrease in calculated creatinine clearance from baseline and urine output less than 0.5 ml/kg/h over 6 h caused by intravenous polymyxin B was defined as nephrotoxicity. Proteinuria, tubular urine or azotemia caused by intravenous polymyxin B was also considered as nephrotoxicity. Skin hyperpigmentation has been also reported in the patients with polymyxin B treatment [17]. The main adverse event of inhaled polymyxin B is bronchospasm.

### Statistical analysis

Data were analyzed using SPSS software, version 22.0 (SPSS). Matched analysis was used. The matching criteria were as follows: age (± 5 years), gender, septic shock, and Apache II score (± 4 points) when started using polymyxin B.

Mean ± standard deviation was used to describe a continuous variable with a normal distribution and Student's t test was used for analysis. Median (interquartile range [IQR]) was used to describe continuous variables without normal distribution and rank-sum test was used to analyze. Categorical variables are expressed as frequency or ratio and analyzed using the χ^2^ test. When appropriate, results were reported as odds ratios (ORs) with associated 95% confidence intervals (CIs). *P* < 0.05 was considered statistically significant. The differences in VAP-related mortality between the IV group and IH plus IV group were assessed using Kaplan–Meier curves. Statistical significance was calculated using the log-rank test of the difference survival curves.

Multivariate logistic regression was used for analyze the effects of polymyxin B usage on the favorable clinical outcomes. The model was generated on the variables with clinical interest and on those with a p value of less than 0.05 in the univariate analysis. The included variables were: age, comorbidities, SOFA score, septic shock, daily dose of IV polymyxin B, treatment with IH + IV polymyxin B and duration of polymyxin B therapy. The effects of polymyxin B usage on the favorable clinical outcomes and on the all-cause of in-hospital mortality were also explored in the following subgroups: SOFA ≥ 8 vs SOFA < 8; surgery admission *vs.* medical admission; with vs without bacteremia; XDR *A. baumannii vs.* XDR *E. coli *vs XDR *K. pneumoniae *vs XDR *P. aeruginosa*; with *vs.* without immunosuppressive status. The cut-off value for SOFA score was determined according to the median value of our patients.

## Results

### Patient demographic and baseline clinical characteristics

**Fig.1:**
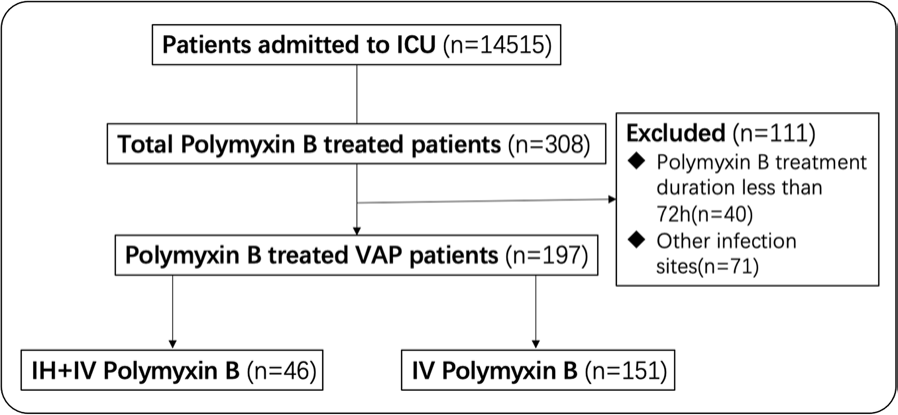
Flowchart of the study inclusion process. VAP: ventilator-associated pneumonia; IH + IV, inhaled and intravenous; IV, intravenous

We included 197 patients with confirmed XDR G- infected VAP, who were admitted to eight tertiary hospitals from January 1st 2018 to January 1st 2020. The study flowchart is shown in Fig. 1. The baseline clinical characteristics and outcomes of the overall population are demonstrated in the Additional file 1: Table S1, S5, respectively.

After matching, there was a total of 132 patients. Among them, 44 patients received simultaneous IH plus IV polymyxin B, and 88 patients received IV polymyxin B alone. Patient demographic and baseline characteristics are shown in Table 1. IH + IV polymyxin B patients were more frequently affected by multiple trauma than IV polymyxin B patients (34% vs 14%, *p* = 0.006). In addition, IH + IV patients had less frequently acute respiratory failure than IV patients (38% vs 47%) although the difference does not reach statistical difference (*p* = 0.06). Moreover, higher rate of IH + IV patients were infected by XDR *K. pneumoniae* than IV patients (70.5% vs 36.4%, *p* < 0.001). All the infecting pathogens had polymyxins MIC value < 1 mg/L (data not shown). The empirical antibiotic therapy before enrollment is shown in the Additional file 1: Table S2. There was no significant difference in the empirical antibiotic therapy between the two groups (*p* = 0.84).

**Table 1:** Demographic and clinical characteristics of study patients after matching

	Total (n = 132)	IH + IV polymyxin B (n = 44)	IV polymyxin B (n = 88)	*p* value
Age, mean years ± SD	65 ± 17	67 ± 17	64 ± 17	0.317
Sex, *n* (%)	103(78.0)	34(77.3)	69(78.4)	0.882
Etiology, *n* (%)				
Acute respiratory failure	54(40.9)	13(29.5)	41(46.6)	0.060
Multiple trauma	27(20.5)	15(34.1)	12(13.6)	0.006
Postoperative	31(23.5)	6(13.6)	25(28.4)	0.059
Acute pancreatitis	12(9.1)	7 (15.9)	6(6.8)	0.108
Others	9 (6.8)	4(9.1)	5(5.7)	0.714
Cause of ICU admission, *n* (%)				
Medical	73(55.3)	23(52.3)	50(56.8)	0.620
Surgical	59(44.7)	21(47.7)	38(43.2)	
Comorbidities, *n* (%)				
Cardiovascular disease	50(37.9)	19(43.2)	31(35.2)	0.374
Cerebrovascular disease	34(25.8)	7(15.9)	27(30.7)	0.067
Chronic pulmonary disease	15(11.4)	5(11.4)	10(11.4)	> 0.999
Chronic liver disease	5(3.8)	1(2.3)	4(4.5)	0.519
Diabetes mellitus	21(15.9)	9(20.5)	12(13.6)	0.313
Chronic kidney disease	20(15.2)	5(11.4)	15(17.0)	0.391
Solid tumor	11(8.3)	5(11.4)	6(6.8)	0.373
Hematological malignancies	7(5.3)	4(9.1)	3(3.4)	0.170
Neutropenia	4(3.0)	2(4.5)	2(2.3)	0.473
HIV	1(0.8)	0(0)	1(1.1)	0.478
Charlson comorbidity index	2 ± 2	2 ± 1	2 ± 2	0.266
Immunosuppressive status, *n* (%)	20(15.2)	9(20.5)	11(12.5)	0.230
Responsible pathogens, *n* (%)				
XDR *Escherichia coli*	4(3.0)	0(0.0)	4(4.5)	0.369
XDR *Klebsiella pneumoniae*	63(47.7)	31(70.5)	32(36.4)	< 0.001
XDR *Acinetobacter baumannii*	53 (40.2)	9(20.5)	44 (50.0)	0.001
XDR *Pseudomonas aeruginosa*	12(9.1)	4(9.1)	8(9.1)	> 0.999
Combination of other antibiotics	76(57.6)	24(54.5)	52(59.1)	0.618
Severity of disease				
SOFA score, mean scores ± SD	8 ± 4	9 ± 4	8 ± 4	0.308
APACHE II score, mean scores ± SD	19 ± 6	18 ± 7	20 ± 5	0.265
Bacteremia, *n* (%)	26(19.7)	8(18.2)	18(20.5)	0.757
Sepsis or septic shock, *n* (%)	74(56.1)	24(54.5)	50(56.8)	0.764
Single maintenance dose of Intravenous polymyxin B, mg/kg, median (IQR)	1.0(1.0, 1.5)	1.0(1.0, 1.25)	1.0(1.0, 1.5)	0.563
Frequency of intravenous polymyxin B, median (IQR)	2.0(2.0,2.0)	2.0(2.0,2.0)	2.0(2.0,2.0)	–
Loading dose of intravenous polymyxin B, mg/kg, median (IQR)	2.0(1.7,2.1)	2.0(1.8,2.1)	2.0(1.7,2.0)	0.652
Dose of inhaled polymyxin B, mg/kg, median (IQR)	–	1.82(1.04,2.0)	–	
Duration of polymyxin B therapy, mean days ± SD	11 ± 8	10 ± 7	12 ± 9	0.090
History of antibiotics within 7 days, *n* (%)	113(85.6)	38(86.4)	75(85.2)	0.861
History of glucocorticoid within 7 days, *n* (%)	41(31.1)	16(36.4)	25(28.4)	0.352
Length of hospitalization, median (IQR)	33(20, 55)	35(23, 55)	33(18, 54)	0.647
Length of ICU stay, median (IQR)	27(16, 41)	25(16, 37)	29(15, 47)	0.808
Duration of mechanical ventilation, median (IQR)	18(11, 30)	18(9, 28)	18(11, 32)	0.827

### Polymyxin B therapy in the matched population

The loading dose of IV polymyxin B was 2.0 (1.7, 2.1) mg/kg. The daily median dose of IV polymyxin B was 2.0 (2.0, 2.5) mg/kg and 2.0 (2.0–3.0) mg/kg in the IH + IV and IV group, respectively. The median daily dose of IH polymyxin B was 1.82 (1.04, 2.0) mg/kg. The IV and IH polymyxin B administered by the center is shown in the Additional file 1: Table S3.

The detailed setting about I/E ratio, respiratory frequency, tidal volume and inspiratory flow by center is shown in Additional file 1: Table S4. In some centers, protective mechanical ventilation strategy was used to treat the severe ARDS patients; to avoid hypercapnia, the respiratory rate was higher than 15/min.

### Outcomes and adverse events in the matched population

**Fig.2:**
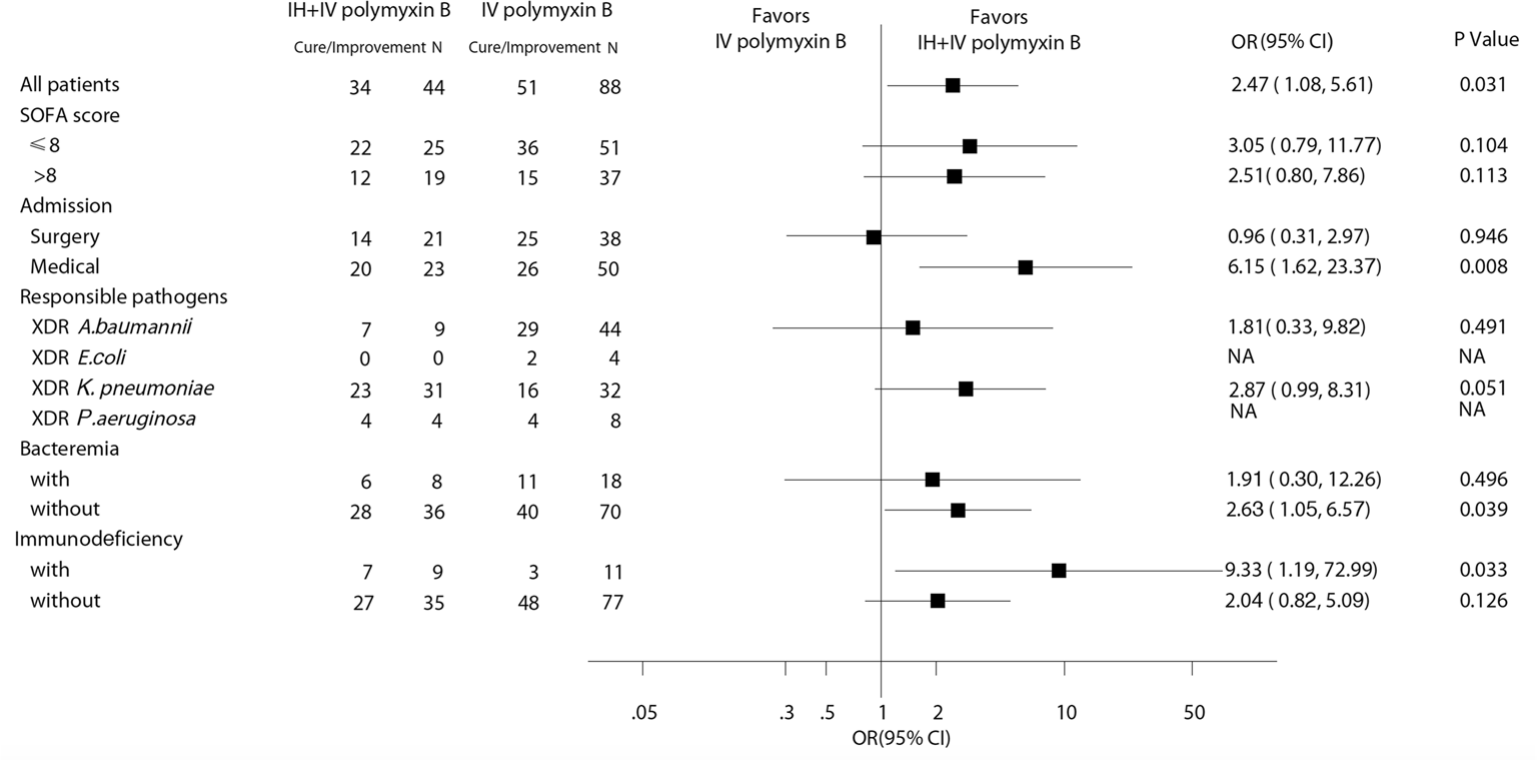
Comparison of clinical cure and improvement in the different subgroups. SOFA, Sequential Organ Failure Assessment; XDR, extensively drug-resistant; A. *baumannii, Acinetobacter baumannii; E. coli, Escherichia coli*; *K. pneumoniae, Klebsiella pneumoniae*; *P. aeruginosa, Pseudomonas aeruginosa*

**Fig.3:**
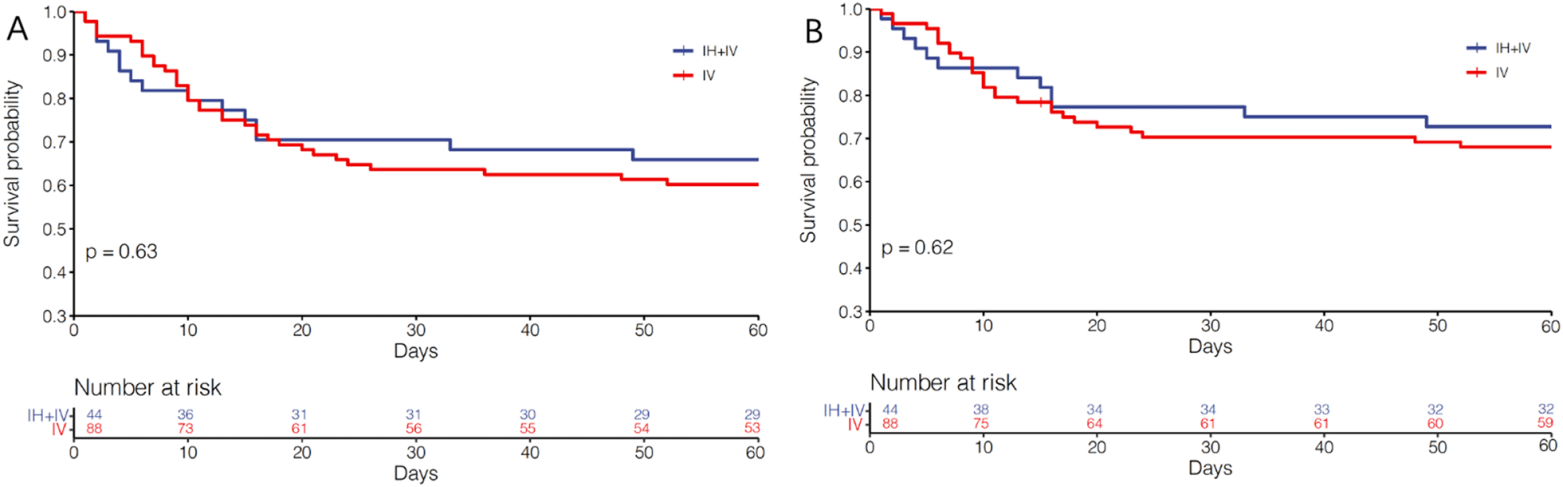
Mortality in two treatment groups in the matched population. **A** All-cause in-hospital mortality; **(B)** VAP-related in-hospital mortality. IH + IV, inhaled plus intravenous; IV, intravenous. Day 0 represent VAP onset

**Fig.4:**
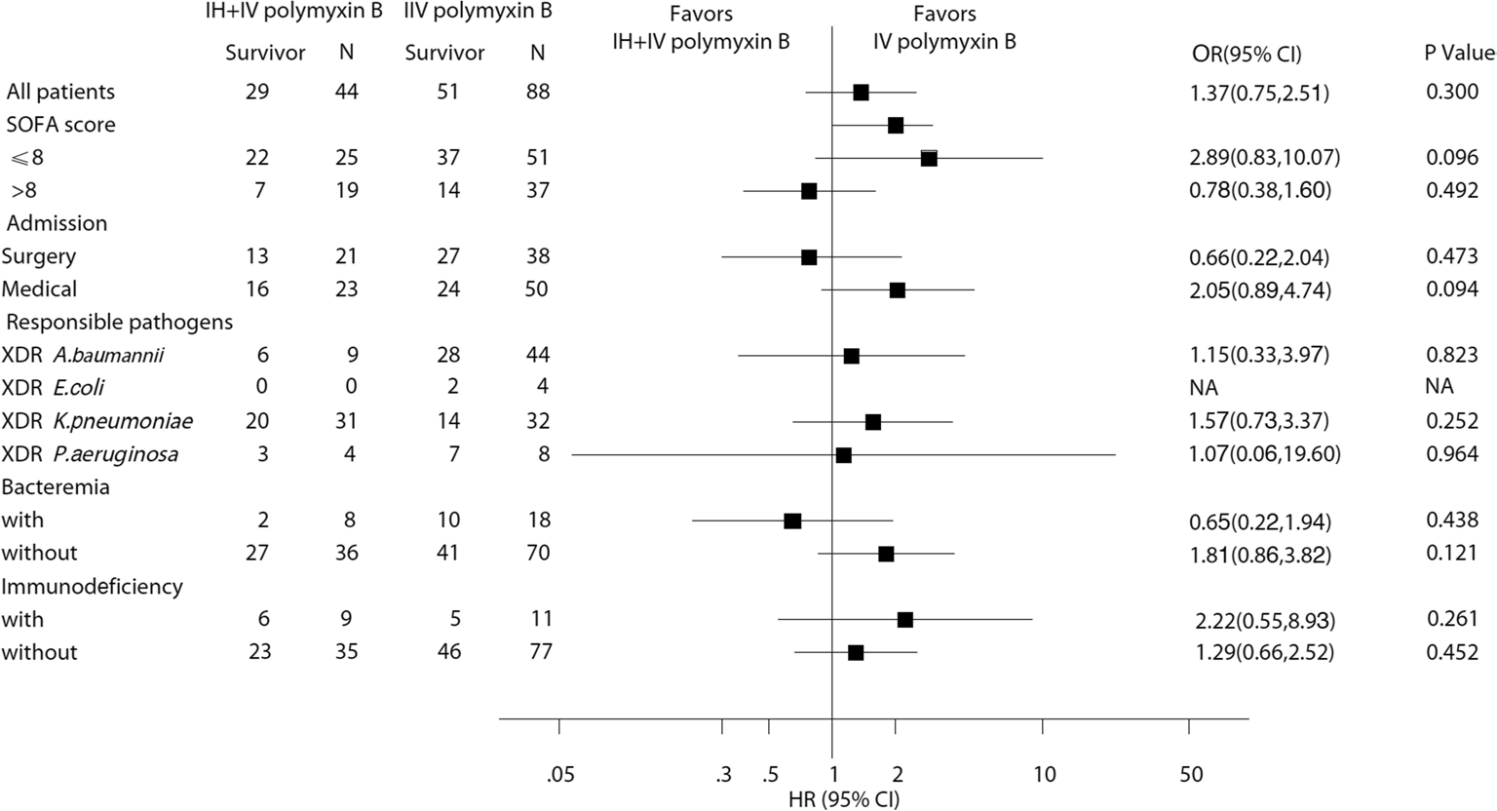
Comparison of all-cause in-hospital mortality in the different subgroups. SOFA, Sequential Organ Failure Assessment; XDR, extensively drug-resistant; A. *baumannii, Acinetobacter baumannii; E. coli, Escherichia coli; K. pneumoniae, Klebsiella pneumoniae; P. aeruginosa, Pseudomonas aeruginosa*

After matching (*n* = 132), the difference in clinical cure rate was not significant between the two groups (43.2% for the IH + IV group vs 27.3% for the IV group, *p* = 0.066). However, a significantly higher rate of favorable clinical outcomes was observed in the IH + IV group compared to the IV group (77.3% vs 58.0%, *p* = 0.029, Table 2). The drug was well tolerated and was associated with slight adverse events. No severe neurotoxicity was observed in all patients, the median AKI classification was stage Injury (Table 2). Patients in the different subgroups (admitted for medical etiology, infected with XDR *K. pneumoniae*, without bacteremia, with immunosuppressive status) were with odds ratios (ORs) substantially favoring additional use of IH polymyxin B (Fig. 2). Secondary endpoints, such as the percentage of microbiological eradication were similar in the two groups (36.4% vs 23.9%, *p* = 0.132). There were no statistically significant differences in all case mortality (*p* = 0.63) or VAP-related mortality (*p* = 0.62) using log-rank test in analysis of Kaplan–Meier curves (Fig. 3A, B). Results of all-cause mortality from various predefined subgroups are reported in Fig. 4. Patients in the different subgroups, including SOFA score ≤ 8 or > 8, surgical or medical admission and different responsible pathogens, with or without bacteremia, with or without immunosuppressive status were without ORs substantially favoring increased all-cause mortality.

**Table 2:** Clinical and bacteriological outcomes, mortality, and adverse events in both treatment groups after matching

	IH + IV polymyxin B (*n* = 44)	IV polymyxin B (*n* = 88)	*p* value
Outcomes			
Clinical outcomes			
Clinical cure	19(43.2)	24(27.3)	0.066
Clinical improvement	15(34.1)	27(30.7)	0.692
Clinical failure	10(22.7)	35(39.8)	0.051
Recurrence	0(0.0)	2(2.3)	0.552
Favorable clinical outcome	34(77.3)	51(58.0)	0.029
Unfavorable clinical outcome	10(22.7)	37(42.0)	
Bacteriological outcomes			
Eradication	16(36.4)	21(23.9)	0.132
Persistence	18(40.9)	45(51.1)	0.267
Colonization	1(2.3)	12(13.6)	0.079
Recurrence	9(20.5)	10(11.4)	0.161
In-hospital mortality			
All-cause	15(34.1)	37(42.0)	0.378
VAP-related	12(27.3)	30(34.1)	0.428
28-day mortality	7(15.9)	22(25.0)	0.234
Side effects			
AKI	3(6.8)	5(5.7)	> 0.999
Bronchospasm	4(9.1)	0	–
Darkening of skin	44(100)	88(100)	–
Facial flushing	14(31.8)	25(28.4)	0.840

### Predictors of favorable clinical outcome in the matched patients with VAP

The univariate analysis of the 132 patients with VAP showed that patients with favorable clinical outcome had a lower SOFA score, a higher daily dose of IV polymyxin B, with the additional use of IH polymyxin B treatment. Outcome has not be improved by the specific antibiotic combination (Table 3).

**Table 3:** Univariate analysis of predictors associated with favorable clinical outcome in 132 patients with VAP

	Unfavorable clinical outcome(*n* = 47)	Favorable clinical outcome(*n* = 85)	*p* value	OR	95% CI
Age, mean years ± SD	68 ± 17	63 ± 17	0.092	0.98	0.96–1.00
Sex, n (%)	37(78.7)	66(77.6)	0.886	0.94	0.40–2.23
Etiology, *n* (%)					
Acute respiratory failure	19(40.4)	35(41.2)	0.933	1.02	0.66–1.57
Multiple trauma	11(23.4)	16(18.8)	0.532	0.80	0.41–1.59
Postoperative	9(19.1)	21(24.7)	0.466	1.29	0.64–2.58
Acute pancreatitis	5(10.6)	7(8.2)	0.646	0.77	0.26–2.30
Others	3(6.4)	6(7.1)	0.883	1.11	0.29–4.22
Cause of ICU admission, *n* (%)					
Medical	27(57.4)	46(54.1)	0.713	0.87	0.43–1.79
Surgical	20(42.6)	39(45.9)	0.811	1.09	0.53–2.24
Comorbidities, *n* (%)					
Cardiovascular disease	23(48.9)	27(31.8)	0.053	0.49	0.23–1.01
Cerebrovascular disease	13(27.7)	21(24.7)	0.710	0.86	0.38–1.92
Chronic pulmonary disease	7(14.9)	8(9.4)	0.346	0.59	0.20–1.76
Chronic liver disease	3(6.4)	2(2.4)	0.264	0.35	0.06–2.20
Diabetes mellitus	9(19.1)	12(14.1)	0.451	0.69	0.27–1.79
Chronic kidney disease	10(21.3)	10(11.8)	0.150	0.49	0.19–1.29
Solid tumor	6(12.8)	5(5.9)	0.180	0.43	0.12–1.48
Hematological malignancies	3(6.4)	4(4.7)	0.682	0.72	0.16–3.38
Neutropenia	2(4.3)	2(2.4)	0.547	0.54	0.07–3.98
HIV	1(2.1)	0(0.0)			
Charlson comorbidity index	2 ± 2	2 ± 2	0.200	0.88	0.73–1.07
Immunosuppressive status, *n* (%)	10(21.3)	10(11.8)	0.150	0.49	0.19–1.29
Responsible pathogens, *n* (%)					
XDR *Escherichia coli*	2(4.3)	2(2.4)	0.936	0.54	0.07–3.98
XDR *Klebsiella pneumoniae*	24(51.1)	39(45.9)	0.568	1.03	0.50–2.13
XDR *Acinetobacter baumannii*	17(36.2)	36(42.4)	0.488	1.10	0.54–2.25
XDR *Pseudomonas aeruginosa*	4(8.5)	8(9.4)	1.000	1.65	0.64–4.26
Combination of other antibiotics	28(59.6)	49(57.6)	0.830	0.92	0.45–1.91
Severity of disease					
SOFA score, median (IQR)	9(6, 13)	6(5, 10)	0.006	0.88	0.81–0.97
APACHE II score, median (IQR)	20(16, 24)	18(14, 23)	0.348	0.97	0.92–1.03
Bacteremia, *n* (%)	9(19.1)	17(20.0)	0.906	1.06	0.43–2.60
Septic shock, *n* (%)	37(78.7)	37(43.5)	0.002	0.30	0.14–0.63
Daily dose of Intravenous polymyxin B, mg/kg, median (IQR)	1.0(1.0, 2.0)	1.0(50, 1.5)	0.015	0.98	0.96–1.00
Frequency of polymyxin B, median (IQR)	2(2,2)	2(2,2)	1	–	–
Duration of polymyxin B therapy, mean days ± SD	11 ± 9	11 ± 7	0.907	1.00	0.95–1.04
History of antibiotics within 7 days, *n* (%)	41(87.2)	72(84.7)	0.692	0.81	0.29–2.29
Treatment with IH + IV polymyxin B, *n* (%)	34(77.3)	10(22.7)	0.031	2.44	1.09–5.56
History of glucocorticoid within 7 days, *n* (%)	14(29.8)	27(31.8)	0.814	1.10	0.51–2.38
Length of hospitalization, median (IQR)	25(15, 40)	37(23, 60)	0.039	–	–
Length of ICU stay, median (IQR)	21(10, 33)	30(17, 48)	0.138	–	–
Duration of mechanical ventilation, median (IQR)	20(13, 30)	16(9, 30)	0.827	–	–
In-hospital mortality					
All-cause	28(59.6)	24(28.2)	0.01		
VAP-related	27(57.4)	15(17.6)	0.001		
28-day mortality	20(42.6)	9(10.6)	0.001		

The logistic regression analysis showed that additional use of IH polymyxin B, presence of sepsis and septic shock and low SOFA score were independent factors associated with favorable clinical outcome (Table 4).

**Table 4:** Logistic regression analysis of predictors associated with favorable clinical outcome in 132 patients with VAP

	Multivariate analysis		
Variables	OR	95% CI	*p* value
IH plus IV polymyxin B	2.63	1.06–6.66	0.037
SOFA score	0.87	0.79–0.96	0.005
Septic shock	0.40	0.18–0.91	0.028

## Discussion

Our multi-center study demonstrated that adjunctive inhalation of polymyxin B did not improve the clinical cure rate in VAP patients caused by XDR GN bacilli. Other secondary endpoints, including all-cause mortality, VAP-related in-hospital, and 28-day mortality and bacteriological outcomes were not different between the two treatment groups. Nevertheless, the additional IH polymyxin B was associated with a more favorable clinical outcome. This might be due to the control of the continuous bacterial contamination of the respiratory tract issued from the endotracheal tube. Low rates of side effects associated with polymyxin B, such as nephrotoxicity and direct toxicity on airways were observed. However, skin hyperpigmentation was observed in almost all the patients treated with polymyxin B.

The incidence of VAP caused by MDR or XDR GN bacilli is continuously increasing. It has been reported that *A. baumannii, K. pneumoniae, and P. aeruginosa* were the most commonly detected pathogens causing VAP [18]. Pathogen resistance to antibiotics has been reported in intensive care units [19]. Our result showed that MDR *K. pneumoniae* was the most common Gram-negative bacilli to cause VAP. The mortality rate of VAP caused by XDR GN bacilli was shown to be high (between 46 and 6%) [20]. In our study, the VAP-related mortality and all-cause mortality rates were consistent with the previous literature [20].

Given that colistimethate sodium have poor penetration into the lung parenchyma but systemic toxicity when administered intravenously, aerosolized and IV colistimethate sodium are commonly combined and administered in the therapy of MDR or XDR GN bacilli VAP [21]. The benefits of aerosolized colistimethate sodium in patients with VAP remain controversial. The retrospective nature of clinical studies, their heterogeneous protocols, the lack of optimization of the technique of nebulization and the variability of dosing restrict the validity of the three meta-analyses published in 2015 and 2018 [10]. There is clinical and experimental evidence that nebulization of high-dose colistimethate sodium may be an efficient treatment of MDR GNB VAP. It is unclear whether nebulized high-dose colistimethate sodium is equivalent or superior to the treatment by intravenous new cephalosporin/ß-lactamase inhibitors. Nebulized polymyxin B was also prescribed to treat MDR bacilli caused HAP or VAP. A single-center retrospective clinical trial aimed at assessing combination of IH and IV polymyxin B treatment on the MDR-*K. pneumoniae* induced VAP patients, found that complete clearance rate of *K. pneumoniae* was higher with the combination of IH and IV polymyxin B compared with IV polymyxin B alone. The average duration of intubation and length of ICU stay were shortened with combined IH and IV polymyxin B therapy [22]. In our study, both the clinical cure and bacteriological clearance were not improved by IH + IV polymyxin B. The presence of sepsis and septic shock, as well as a high SOFA score may significantly affect outcome. The discrepancy in the findings from the current study and the previous trial may be related to differences in the doses and in the studied populations. In the current study, the doses of intravenous and nebulized polymyxin B were relatively lower, and the types of the pathogens were multiple. The recommendation of IV polymyxin B dose is 50–100 mg/day in the < Instruction > in China [23]. However, this recommended dose is not based on weight and could be inefficient in the case of high weight. It was found that IV doses of polymyxin *B* < 200 mg/day cannot achieve bactericidal efficiency [24, 25]. Therefore, to achieve bactericidal efficiency, the international guideline recommended that for patients with severe infections, a polymyxin B dose of 1.25–1.5 mg/kg every 12 h is infused over 1 h preceded by a loading dose of 2.0–2.5 mg/kg over 1 h [26]. However, a recent Chinese study showed that over 0.75 mg/kg/12 h IV polymyxin B achieved a cumulative fraction of response > 90% for MDR *A. baumannii, K. pneumoniae* and *P. aeruginosa* induced bloodstream infection patients (MICs ranging from 0.125 to > 32 mg/L) [27]. To remedy the poor diffusion of systematic polymyxin B in the pulmonary, the appropriate IH dose was important. It was demonstrated that nebulized dose of polymyxin B was as high as 8.24 mg/kg in the neutropenic mouse model to achieve the AUC/MIC targets associated with bacteriostasis against *P. aeruginosa* [28]. The optimal nebulized dose of polymyxin B is essentially determined by its bronchial tolerability and the extrapulmonary deposition during the nebulization phase. In the Chinese expert consensus of clinical polymyxin B use, the recommended IH dose is 50 mg, twice a day [29]. A recent study demonstrated that HAP or VAP patient with MDR *K. pneumoniae* treated with 2.0–2.5 mg/kg/day with or without additional IH polymyxin B at 100 mg/day, a complete clearance of *K. pneumoniae* was 92.1% in the IH and IV group compared to 70.1% in the IH group [30]. Thus, we found reasonable to get the current result for clinical cure rate of 27%, a bacteriological eradication of 24%. In the present study, the dose of polymyxin B was lower than international recommendation in several centers. With the increasing use of polymyxin B, the reliable PK data of nebulized polymyxin B to optimize the dosage selection in the critical illness are needed in the future.

The favorable clinical outcome rate in the IH + IV polymyxin B group was significantly higher than that in IV polymyxin B group. A previous study from a small size study conducted by Sobieszczyk et al. showed that 19/25 of patients treated with polymyxin B-based therapy had a favorable response [31], which was consistent with findings of our study. A possible hypothesis for improved favorable outcome was related to a partial control of the continuous bacterial contamination of the respiratory tract coming from the endotracheal tube (reduction of the inoculum). Another possible interpretation of the more favorable clinical outcome was that more multiple trauma and less acute respiratory failure patients in the IV + IH polymyxin B group than in the IV polymyxin B group. Since it was well known that outcome of trauma patients is better than medical critically ill patients. In addition, acute respiratory failure was an independent risk factor for mortality of critically ill patients [32].

The common side effect of inhaled antibiotics was bronchospasm. Bronchospasm was found in three patients with IH + IV polymyxin B treatment (6.8%). Bronchospasm caused by polymyxin B inhalation in those three patients was not permanent and was consequently relieved by bronchodilators and glucocorticoids. Moreover, the incidence of nephrotoxicity was 6.1% (8/132), which was similar with that reported in previous studies (6%–10%) [31]. Compared with IV polymyxin B, the combination of IH + IV polymyxin B did not further increase nephrotoxicity. So polymyxin B may be used instead when the lack of colistimethate sodium or patients with severe kidney dysfunction.

Skin hyperpigmentation in the head and neck was observed in almost all the patients treated with polymyxin B. It is possible that skin darkening was associated with the release of histamine caused by polymyxin B, as histamine is known to exert melanogenic effects [33]. We found that skin darkening could be reversed without any treatment within 6 months by the end of polymyxin B treatment.

To the best of our knowledge, this is the largest matched cohort study reporting the benefit of IH in addition to IV polymyxin B in patients with VAP caused by XDR GN bacilli. However, this study has some limitations. First, the intravenous and nebulization doses of polymyxin B was relatively lower in several centers, which may decrease the bacteria clearance and clinical outcomes. Second, this is a retrospective study with a relatively low sample size. However, it should be considered that polymyxin B has only recently become available in China. Third, our primary outcome was a subjective one. However, we had two blinded investigators who did not know the groups to determine the primary results to avoid inherent bias. Fourth, a large number of patients could not be matched, which is a potential source of bias. Fifth, despite matching on four factors, both groups were different concerning the severity of disease: in patients with more favorable clinical outcome, SOFA score and the incidence of septic shock were significantly lower using multivariate analysis. These results suggest that the more favorable clinical outcome was not related to treatment of ventilator-associated pneumonia but to differences in the severity of the underlying disease. Using propensity score, a more accurate statistical method for matching, would have likely concealed this important result that explains the apparent contradiction between the lack of therapeutic effect of low-dose IH + IV polymyxin for treating VAP and a more favorable outcome. Finally, the present study was a multi-center clinical trial, but data were collected retrospectively. The procedure for nebulization was not strictly identical in all the centers.

To summarize, our data indicated that in patients with VAP caused by XDR GN bacilli, the addition of IH polymyxin B to IV polymyxin B did not provide efficient clinical cure and bacterial eradication compared to IV polymyxin B alone. However, it may provide a more favorable clinical outcome, maybe due to the control of the continuous bacterial contamination of the respiratory tract issuing from the endotracheal tube (reduction of the inoculum).

## Supplementary Information

**Additional file 1: Table S1** Demographic and Clinical Characteristics of Study Patients. **Table S2** Empirical antibiotic therapy before enrolment. **Table S3** Intravenous and nebulized doses of polymyxin B by center. **Table S4** The information for nebulization of Polymyxin B in each center. **Table S5** Clinical and Bacteriological Outcomes, Mortality, and Adverse Events in Both Treatment Groups.
