# Supplementary material for: Low-dose intravenous plus inhaled versus intravenous polymyxin B for the treatment of extensive drug-resistant Gram-negative ventilator-associated pneumonia in the critical illnesses: a multi-center matched case–control study
Source: Ann Intensive Care. 2022 Aug 8;12:72. doi: 10.1186/s13613-022-01033-5 (PMC9357592; doi:10.1186/s13613-022-01033-5)
Supplement: Supplementary file 1 — Additional file 1: Table S1 Demographic and Clinical Characteristics of Study Patients. Table S2 Empirical antibiotic therapy before enrolment. Table S3 Intravenous and nebulized doses of polymyxin B by center. Table S4 The information for nebulization of Polymyxin B in each center. Table S5 Clinical and Bacteriological Outcomes, Mortality, and Adverse Events in Both Treatment Groups. [file 13613_2022_1033_MOESM1_ESM.docx]

**Additional file Table S1 Demographic and Clinical Characteristics of Study Patients**

|  | Total  (n=197) | IH+IV polymyxin B  (n=46) | IV polymyxin B  (n=151) | *p* value |
| --- | --- | --- | --- | --- |
| Age, median years (IQR) | 61 (50, 73) | 69 (59, 78) | 59 (50, 71) | 0.017 |
| Sex, male (%) | 152 (77.2) | 35 (76.1) | 117 (77.5) | 0.999 |
| Etiology, n (%) |  |  |  |  |
| Acute respiratory failure | 130 (66) | 19 (41.3) | 111 (73.5) | < 0.001 |
| Multiple Trauma | 35 (17.8) | 16 (34.8) | 19 (12.6) | 0.001 |
| Postoperative | 65 (33) | 8 (17.4) | 57 (37.7) | 0.017 |
| Acute pancreatitis | 17(8.6) | 8 (17.4) | 9 (6.0) | 0.016 |
| Others | 16(8.1) | 6 (13.0) | 10 (6.6) | 0.163 |
| Cause of ICU admission, n (%) |  |  |  |  |
| Medical | 112 (56.9) | 23 (50) | 89 (58.9) | 0.367 |
| Surgical | 84 (42.6) | 23 (50) | 61 (40.4) | 0.326 |
| Comorbidities, n (%) |  |  |  |  |
| Cardiovascular disease | 66 (33.5) | 19 (41.3) | 47 (31.1) | 0.270 |
| Cerebrovascular disease | 52 (26.4) | 7 (15.2) | 45 (29.8) | 0.076 |
| Chronic pulmonary disease | 22 (11.2) | 5 (10.9) | 17 (11.3) | 0.942 |
| Chronic liver disease | 6 (3.0) | 1 (2.2) | 5 (3.3) | 0.990 |
| Diabetes mellitus | 34 (17.3) | 9 (19.6) | 25 (16.6) | 0.803 |
| Chronic kidney disease | 26 (13.2) | 5 (10.9) | 21 (13.9) | 0.776 |
| Solid tumor | 18 (9.1) | 5 (10.9) | 13 (8.6) | 0.770 |
| Hematological malignancies | 11 (5.6) | 4 (8.7) | 7 (4.6) | 0.287 |
| Neutropenia | 5 (2.5) | 2 (4.3) | 3 (2) | 0.332 |
| HIV | 5 (2.5) | 0 (0) | 5 (3.3) | 0.592 |
| Charlson comorbidity index | 2 (0, 3) | 2 (1, 3) | 2 (0, 3) | 0.734 |
| Immunosuppressive status, n (%) | 37(18.9) | 20(43.5) | 17(11.3) | <0.001 |
| Responsible Pathogens, n (%) |  |  |  |  |
| XDR *Escherichia coli* | 7 (3.6) | 1 (2.2) | 6 (4.0) | 0.564 |
| XDR *Klebsiella pneumoniae* | 83(42.1) | 31(67.4) | 52(34.4) | <0.001 |
| XDR *Acinetobacter baumannii* | 75(38.1) | 9(19.6) | 66(43.7) | 0.027 |
| XDR *Pseudomonas aeruginosa* | 32(16.2) | 5(10.9) | 27 (17.9) | 0.259 |
| Combination of other antibiotics | 87(44.2) | 24(52.2) | 63(41.7) | 0.211 |
| Severity of disease |  |  |  |  |
| SOFA score, median scores (IQR) | 9 (5, 12) | 8.5 (5, 12) | 9 (5, 12) | 0.658 |
| APACHE II score, median scores (IQR) | 21 (16, 27) | 18.5 (14, 25) | 22 (17.5, 28) | 0.005 |
| Bacteremia, n (%) | 40 (20.3) | 8 (17.4) | 32 (21.2) | 0.725 |
| Sepsis or Septic shock, n (%) | 111 (56.3) | 16 (34.8) | 95 (62.9) | 0.001 |
| Dose of Intravenous polymyxin B, mg/kg, median (IQR) | 1.0(1.0, 1.5) | 1.0(1.0, 1.25) | 1.0(1.0, 1.5) | 0.563 |
| Duration of polymyxin B therapy, median days (IQR) | 8 (5, 11) | 8 (4, 9) | 8 (5, 12) | 0.097 |
| History of antibiotics within 7 days, n (%) | 169 (85.8) | 41 (89.1) | 128 (84.8) | 0.617 |
| History of Glucocorticoid within 7 days, n (%) | 67(34.0) | 16 (34.8) | 51 (33.8) | 0.900 |
| Length of hospitalization, median (IQR) | 31 (19, 53) | 31 (23, 53) | 31 (19, 52) | 0.459 |
| Length of ICU stay, median (IQR) | 23 (12, 40) | 22 (12, 37) | 23 (13, 45) | 0.527 |
| Duration of mechanical ventilation, median (IQR) | 14 (5, 26) | 16 (5, 27) | 14 (6, 26) | 0.634 |

**Additional file Table S2** **Empirical antibiotic therapy before enrollment**

| **Variables** | **Total**  **(n = 132)** | **IV polymyxin B**  **(n = 88)** | **IH+IV polymyxin B  (n = 44)** | **p** |
| --- | --- | --- | --- | --- |
| Antibiotics, n (%) | | | | |
| Enzyme Inhibitors | 13 (10) | 9 (10) | 4 (9) | 0.1 |
| Quinolones | 1 (1) | 1 (1) | 0 (0) |  |
| Aminoglycosides | 2 (2) | 0 (0) | 2 (5) |  |
| Carbapenem | 23 (17) | 18 (20) | 5 (11) |  |
| Others | 8 (6) | 3 (3) | 5 (11) |  |
| Combined medication | 85 (64) | 57 (65) | 28 (64) |  |
| Empirical antibiotic failure, n (%) | | | | |
| No | 93 (70) | 63 (72) | 30 (68) | 0.84 |
| Yes | 39 (30) | 25 (28) | 14 (32) |  |

**Additional file Table S3 Intravenous and nebulized doses of polymyxin B by center**

| **Center** | **IV loading dose(mg/kg)** | **IV maintenance dose (mg/kg)** | **IV frequency** | **Daily IV dose (mg/kg)** | **IV Duration**  **(days)** | **Single IH dose (mg/kg)** | | **IH frequency** | | **Daily IH dose (mg/kg)** | **IH Duration**  **(days)** |
| --- | --- | --- | --- | --- | --- | --- | --- | --- | --- | --- | --- |
| 1 | 2.00(1.96,2.08) | 1.04(1.00,1.33) | 2(2,2) | 2.08(2.00,2.66) | 7.5(4.5,10.0) | 1.00(0.76,1.02) | | | 2(2,2) | 2.00(1.52,2.04) | 7.5(4.5,10.0) |
| 2 | 1.87(1.56,2.00) | 1.11(1.00,1.17) | 2(2,2) | 2.23(2.00,2.34) | 12.0(8.0,14.5) | 1.00(1.00,1.02) | | | 2(2,2) | 2.00(2.00,2.04) | 12.0(8.0,14.5) |
| 3 | 1.86(1.68,2.00) | 1.02(1.00,1.13) | 2(2,2) | 2.04(2.00,2.25) | 9.0(5.5,9.0) | 0.83(0.50,0.86) | | | 2(2,2) | 1.67(1.00,1.72) | 9.0(5.5,9.0) |
| 4 | 1.67(1.67,1.92) | 1.25(1.15,1.25) | 2(2,2) | 2.50(2.30,2.50) | 10.0(7.0,12.0) | 1.25(1.18,1.26) | | | 2(2,2) | 2.50(2.36,2.52) | 10.0(7.0,12.0) |
| 5 | 2.08(2.00,2.17) | 1.09(1.02,1.17) | 2(2,2) | 2.17(2.04,2.34) | 7.0(5.0,11.0) | 0.54(0.50,1.01) | | | 2(2,2) | 1.08(1.00,2.02) | 7.0(5.0,11.0) |
| 6 | 1.92(1.92,1.96) | 0.98(0.96,1.04) | 2(2,2) | 1.96(1.92,2.08) | 11.0(8.0,11.0) | 0.96(0.89,0.98) | | | 2(2,2) | 1.92(1.79,1.96) | 11.0(8.0,11.0) |
| 7 | 2.00(2.00,2.04) | 1.00(1.00,1.04) | 2(2,2) | 2.00(2.00,2.08) | 11.0(7.0,11.0) | 0.66(0.58,0.79) | | | 2(2,2) | 1.33(1.17,1.58) | 11.0(7.0,11.0) |
| 8 | 1.81(1.81,1.92) | 0.91(0.91,0.96) | 2(2,2) | 1.82(1.82,1.92) | 7.0(5.0,8.0) | | 0.91(0.91,0.96) | | 2(2,2) | 1.82(1.82,1.92) | 7.0(5.0,8.0) |

**Center 1:**The First Affiliated Hospital of Anhui Medical University; **Center 2:**The First Affiliated Hospital of Nanchang University; **Center 3:** The first people’s Hospital of KunShan; **Center 4:** Lishui People's Hospital; **Center 5**: Ruijin Hospital, Shanghai Jiao Tong University School of Medicine; **Center 6:** Lishui People's Hospital; **Center 7:** Lishui People's Hospital; **Center 8:** Zhejiang Hospital

**Additional file Table S4 The information for nebulization of Polymyxin B in each center**

| **Center** | **Mode of mechanical ventilation** | **I/E ratio** | **end-inspiratory pause duration(s)** | **Respiratory frequency**  **(min^-1^)** | **Tidal volume**  **(ml/kg)** | **Inspiratory flow rate**  **(L/min)** |
| --- | --- | --- | --- | --- | --- | --- |
| 1 | SIMV PC+PS | NA | NA | 15(15,15) | NA | NA |
| 2 | Volume-controlled mode | 1:2.30  (1:2.25,1:2.32) | 0.2(0.2,0.2) | 15(15,15) | 8(8,8) | 30(30,33) |
| 3 | Volume-controlled mode | 1:2.33  (1:2.30,1:2.33) | 0.2(0.2,0.2) | 15(15,15) | 8(8,8) | 30(30,30) |
| 4 | NA | NA | NA | NA | NA | NA |
| 5 | Volume-controlled mode | 1:2.33  (1:2.20,1:2.33) | 0.2(0.2,0.2) | 15(15,18) | 8(8,8) | 30(30,30) |
| 6 | Volume-controlled mode | 1:2.26  (1:2.17,1:2.33) | 0.2(0.2,0.2) | 15(15,16) | 8(6,8) | 30(30,30) |
| 7 | Volume-controlled mode | 1:2.26  (1:2.17,1:2.33) | 0.2(0.2,0.2) | 15(15,16) | 8(8,8) | 30(30,30) |
| 8 | Volume-controlled mode | 1:2.26  (1:2.17,1:2.33) | 0.2(0.2,0.2) | 15(15,16) | 8(8,8) | 30(30,30) |

**Center 1:**The First Affiliated Hospital of Anhui Medical University, the matched patients used SIMV(PC)+PS model (n=3); **Center 2:**The First Affiliated Hospital of Nanchang University; **Center 3:** The first people’s Hospital of KunShan; **Center 4:** Lishui People's Hospital, the information for nebulization were missing(n=3); **Center 5**: Ruijin Hospital, Shanghai Jiao Tong University School of Medicine; **Center 6:** Lishui People's Hospital; **Center 7:** Lishui People's Hospital; **Center 8:** Zhejiang Hospital

**Additional file Table S5 Clinical and Bacteriological Outcomes, Mortality, and Adverse Events in Both Treatment Groups**

|  | IV polymyxin B (n=151) | | IH+IV polymyxin B  (n=46) | | | *p* value |
| --- | --- | --- | --- | --- | --- | --- |
| Outcomes |  |  | | |  | |
| Clinical outcomes n (%) |  |  | | |  | |
| Clinical cure | 50 (33.1) | | | 19 ( 41.3) | 0.399 | |
| Clinical improvement | 39 (25.8) | | | 17 ( 37.0) | 0.201 | |
| Clinical failure | 59 (39.1) | | | 10 ( 21.7) | 0.048 | |
| Recurrence | 3 ( 2.0) | | | 0 ( 0.0) | 0.783 | |
| Favorable or unfavorable clinical outcome |  | | |  |  | |
| Favorable clinical outcome | 89 (58.9) | | | 36 ( 78.3) | 0.027 | |
| Unfavorable clinical outcome | 62 (41.1) | | | 10 ( 21.7) | 0.026 | |
| Bacteriological outcomes |  | | |  |  | |
| Eradication | 43 (28.5) | | | 18 ( 39.1) | 0.236 | |
| Persistence | 75 (49.7) | | | 18 ( 39.1) | 0.278 | |
| Colonization | 15 ( 9.9) | | | 1 ( 2.2) | 0.168 | |
| Recurrence | 18 (11.9) | | | 9 ( 19.6) | 0.282 | |
| In-hospital Mortality |  | | |  |  | |
| All-cause | 15 (32.6) | | | 43 (28.5) | 0.724 | |
| VAP-related | 12 (26.1) | | | 31 (20.5) | 0.552 | |
| 28-day Mortality | 7 (15.2) | | | 39 (25.8) | 0.197 | |
| Side Effects |  | | |  |  | |
| AKI | 7 (4.6) | | | 3 (6.5) | 0.701 | |
| Bronchospasm | 0 | | | 4(9.1) | NA | |
| Darkening of skin | 151(100) | | | 46(100) |  | |
